# Supplementary material for: On the optimal presence strategies for workplace during pandemics: A COVID-19 inspired probabilistic model
Source: PLoS One. 2023 May 12;18(5):e0285601. doi: 10.1371/journal.pone.0285601 (PMC10180602; doi:10.1371/journal.pone.0285601)
Supplement: Attachment — Submitted filename: Response to Reviewer #2.pdf [file pone.0285601.s002.pdf]

# On the optimal presence strategies for workplace during pandemics: A COVID-19 inspired probabilistic model

**PONE-D-23-00247**

Detailed Response to Reviewer

**Dear Reviewer,**

We appreciate your time and consideration to review our revised manuscript. We applied your comments and those of the two other reviewers to our revised draft of the paper. We highlighted the major parts with colored text in the revised manuscript. Additionally, you can find a point-to-point response to them in the following. Comments (**C**) and Answers (**A**)

**Reviewer #2:** The authors developed a model to compute the optimal trade-off solutions for minimizing the risk of infection and maximizing the productivity in companies during the COVID-19 pandemic. They proposed a probabilistic analysis approach to compute the expected number of infected employees over the time by incorporating basic influential parameters such as the local incidence level, number of contacts among the employees and their average test interval and vaccination rate. The paper is good but needs some improvements. In the **introduction**, it should focus more on the objective of this work and the **importance** of it, and why it is essential for its contribution to the literature.

More updated references (year 2021, 2021, 2022, 2033) should be added to the literature section. Recommendations should also be made in the last section of the text. For companies, adopting a Sustainable Development Goals (SDGs) approach is very important in the aftermath of the Covid-19 pandemic. The importance of the SDGs in business should be mentioned as a major future implication. Here are some references you can cite and include:
Cifuentes-Faura, J. (2022). Circular economy and sustainability as a basis for economic recovery post-COVID-19. Circular Economy and Sustainability, 2(1), 1-7.
Van Zanten, J. A., & Van Tulder, R. (2020). Beyond COVID-19: Applying "SDG logics" for resilient transformations. Journal of International Business Policy, 3(4), 451-464.
Cifuentes-Faura, J. (2021). COVID-19 and the opportunity to create a sustainable world through economic and political decisions. World Journal of Science, Technology and Sustainable Development, 18(4), 417-421.
Mattera, M., Gonzalez, F. S., Ruiz-Morales, C. A., & Gava, L. (2021). Facing a global crisis-how sustainable business models helped firms overcome COVID. Corporate Governance: The International Journal of Business in Society.

**Response:** Thank you for these comments. To address them, we reorganized the Introduction and Related Work sections, and added updated references in addition to those you suggested. We also implemented further changes, mostly adding explanations to clarify and highlight the motivation and contribution of the manuscript, to the Abstract and Conclusion sections.
