# Supplementary material for: On the optimal presence strategies for workplace during pandemics: A COVID-19 inspired probabilistic model
Source: PLoS One. 2023 May 12;18(5):e0285601. doi: 10.1371/journal.pone.0285601 (PMC10180602; doi:10.1371/journal.pone.0285601)
Supplement: Attachment — Submitted filename: Response to Reviewers_JMC.pdf [file pone.0285601.s003.pdf]

On the optimal presence strategies for workplace during pandemics: A COVID-19 inspired probabilistic model

PONE-D-23-00247R1

Detailed Response to Reviewer

Dear Reviewer,

Once again, we appreciate your effort to review our manuscript. We applied the comments, and you can find a point-to-point response to them in the following.

Review Comments to the Author

Please use the space provided to explain your answers to the questions above. You may also include additional comments for the author, including concerns about dual publication, research ethics, or publication ethics. (Please upload your review as an attachment if it exceeds 20,000 characters)

Reviewer #1: well done

Reviewer #2: The manuscript has improved a lot. I only recommend that authors improve the quality of figures before publication.

Response:

We have used a digital diagnostic tool, *Preflight Analysis and Conversion Engine* (PACE), to check and correct the figures. This tool is recommended by PLOS ONE journal at this link: https://pacev2.apexcovantage.com/.

Now in the revision, all the uploaded figures are the output of PACE, and satisfy the journal's requirements.

With many thanks,
The Authors
